# High-resolution macromolecular crystallography at the FemtoMAX beamline with time-over-threshold photon detection

**DOI:** 10.1107/S1600577520014599

**Published:** 2021-01-01

**Authors:** Maja Jensen, Viktor Ahlberg Gagnér, Juan Cabello Sánchez, Åsa U. J. Bengtsson, J. Carl Ekström, Tinna Björg Úlfarsdóttir, Maria-Jose Garcia-Bonete, Andrius Jurgilaitis, David Kroon, Van-Thai Pham, Stefano Checcia, Hélène Coudert-Alteirac, Siawosch Schewa, Manfred Rössle, Helena Rodilla, Jan Stake, Vitali Zhaunerchyk, Jörgen Larsson, Gergely Katona

**Affiliations:** a Department of Chemistry and Molecular Biology, University of Gothenburg, Gothenburg, Sweden; b Department of Microtechnology and Nanoscience, Chalmers University of Technology, Gothenburg, Sweden; c Department of Physics, Lund University, PO Box 118, Lund 22100, Sweden; d MAX IV Laboratory, Lund University, PO Box 118, Lund 22100, Sweden; e Center for Quantum Electronics, Institute of Physics, Vietnam Academy of Science and Technology, Hanoi, Vietnam; f Technische Hochschule Lübeck, Lübeck, Germany; g Department of Physics, University of Gothenburg, Gothenburg, Sweden

**Keywords:** time-over-threshold, femtosecond, multilayer monochromator, macromolecular crystallography

## Abstract

Protein dynamics contribute to protein function on different time scales. Ultrafast X-ray diffraction snapshots can visualize the location and amplitude of atom displacements after perturbation. Since amplitudes of ultrafast motions are small, high-quality X-ray diffraction data is necessary for detection. Diffraction from bovine trypsin crystals using single femtosecond X-ray pulses was recorded at FemtoMAX, which is a versatile beamline of the MAX IV synchrotron. The time-over-threshold detection made it possible that single photons are distinguishable even under short-pulse low-repetition-rate conditions. The diffraction data quality from FemtoMAX beamline enables atomic resolution investigation of protein structures. This evaluation is based on the shape of the Wilson plot, cumulative intensity distribution compared with theoretical distribution, *I*/σ, *R*_merge_/*R*_meas_ and CC_1/2_ statistics versus resolution. The FemtoMAX beamline provides an interesting alternative to X-ray free-electron lasers when studying reversible processes in protein crystals.

## Abstract 2

A 1.5 Å resolution crystal structure of bovine trypsin at room temperature has been determined using 150 fs X-ray diffraction snapshots at the FemtoMAX beamline of the MAX IV facility.

## 1. Introduction   

X-ray diffraction studies are performed either with monochromatic or polychromatic X-rays. Laue diffraction occurs when polychromatic X-ray beams are used. With both methods, it is necessary to obtain several projections of the diffraction pattern with different orientations of the crystal or crystals. The crystal is either rotated using the oscillation methods or kept still during the recording of the image. Laue diffraction visualizes a wider segment of the reciprocal lattice, therefore traditionally the crystal is held still (Wöhri *et al.*, 2010 ▸; Srajer *et al.*, 1996 ▸). Static monochromatic diffraction experiments have a long history, but they were superseded by oscillation experiments on single crystals (Otwinowski, 1993 ▸; Otwinowski & Minor, 1997 ▸). Even though static, monochromatic diffraction images reveal only a thin slice of the reciprocal lattice, it is still possible to obtain high-quality crystal structures through averaging many partially recorded reflections (Sharma *et al.*, 2017 ▸). This shotgun approach emerged with the development of serial crystallography where often only one projection is available from each randomly oriented crystal, either because the crystal is destroyed during recording [serial femtosecond crystallography at X-ray free-electron lasers (XFELs)] (Chapman *et al.*, 2011 ▸; Boutet *et al.*, 2012 ▸; Redecke *et al.*, 2013 ▸) or because an irreversible chemical or physical process renders it unusable for further analysis (capturing snapshots of irreversible reactions) (Schulz *et al.*, 2018 ▸). Sparse static projections contain partially recorded reflections, which make the inference of reflection intensity more difficult. Modern synchrotron facilities provide very high beam intensities, which makes it difficult to obtain multiple projections before the crystal is substantially damaged. Synchrotron-based serial crystallography can mitigate both the radiation damage issues associated with intense beams and open up new opportunities for studying irreversible reactions in crystals on a wide range of time scales (Schulz *et al.*, 2018 ▸; Lan *et al.*, 2018 ▸). The emergence of serial crystallography stirred up many of the standard practices and there is a resurgence of creative efforts for improving data analysis (Sharma *et al.*, 2017 ▸; Brewster *et al.*, 2018 ▸; Fewster, 2018 ▸; Zwart & Perryman, 2020 ▸). Nevertheless, a vast majority of routine protein crystallographic experiments are still performed with the oscillation method using monochromatic X-ray beams (Dauter, 2017 ▸).

Laue diffraction has a natural advantage when snapshots of the crystal structure is required. In time-resolved diffraction experiments, there is usually not enough time for rotating the crystal to obtain the diffraction pattern (Srajer *et al.*, 1996 ▸). An additional advantage of polychromatic experiments is that the number of photons typically exceeds that of monochromatic beams. This advantage however is eclipsed by the brightness of modern synchrotron radiation facilities, where the monochromatic X-ray intensities are already highly damaging for protein crystals (Yamamoto *et al.*, 2017 ▸). The disadvantages of Laue diffraction are the spatial and harmonic overlaps of reflections if the bandwidth of the X-rays is large, and the necessity of specialized diffraction analysis software such as *lauegen* (Campbell *et al.*, 1998 ▸), *Precognition* ^TM^ (Renz Research, Inc.) and *TRex* (Schotte *et al.*, 2013 ▸). Stationary crystals are not required for interpreting Laue diffraction: oscillation experiments were shown to work when using a multilayer monochromator in conjunction with data analysis software designed for monochromatic diffraction (Otwinowski & Minor, 2001 ▸; Deacon *et al.*, 1998 ▸).

The femtosecond X-ray beamline at the MAX IV short-pulse facility (SPF) generates very short and suitably intense X-ray pulses (Enquist *et al.*, 2018 ▸; Larsson *et al.*, 2018 ▸). The pulse length is approximately 100 fs at wavelengths matching inter-atomic distances (Å). X-ray free-electron lasers produce X-ray pulses with similar pulse lengths at much higher intensity. This does not automatically translate into higher-resolution protein crystal structures. At the time of writing, the Protein Data Bank contains only 11 crystal structures with higher than 1.5 Å resolution from XFEL facilities.

Here, we use an effective data collection strategy with a less intense femtosecond X-ray source. This work is a step towards performing time-resolved experiments on protein crystals at the FemtoMAX beamline. We rotate the crystal stepwise around a single axis in a controlled manner resulting in multiple stationary projections. We irradiate the crystal with X-rays produced by a multilayer monochromator and treat the resulting streaky diffraction patterns with a software originally designed for monochromatic diffraction and oscillation data collection (*XDS*) (Kabsch, 2010 ▸). An untested aspect of this work is the use of time-over-threshold (ToT) detector mode of the pixel array detector, for recording diffraction intensities from protein crystals (Enquist *et al.*, 2018 ▸). We show that it is possible to model individual isotropic *B*-factors of atoms based on the protein diffraction data obtained at the FemtoMAX beamline.

## 2. Materials and methods   

### 2.1. Protein crystallization   

Bovine trypsin (Sigma) was dissolved in 30 m*M* HEPES pH 7.0, 3 m*M* calcium chloride and 6 mg ml^−1^ benzamidine to obtain a 60 mg ml^−1^ protein solution. Crystals were obtained using the hanging drop vapor diffusion method, 5 µl protein solution was mixed with 5 µl of precipitant solution (18% PEG8000, 50 m*M* HEPES pH 7.0, 0.2 *M* ammonium sulfate, 3 m*M* calcium chloride and 6 mg ml^−1^ benzamidine).

#### 2.1.1. X-ray diffraction data collection at the FemtoMAX beamline   

The crystals were held at room temperature in the MiTiGen plastic capillaries with 1 µl mother liquor at the end of the capillary maintaining constant vapor pressure over the crystal. Glass or quartz can be used as alternative sealing materials to prevent the capillary content from drying out. The choice of material is also influenced by its optical properties since the pump pulse may be absorbed or reflected and cannot reach the crystal. In addition, the sealing material affects the X-ray diffuse scattering background and generally amorphous sealing material containing lighter elements are preferred. The capillary was sealed with vacuum grease at the base of the pin. A Huber one-circle goniometer base, model 411 X2 W1, was used as crystal rotation stage. 100 images were collected at every 0.1° rotation. 150 fs X-ray pulses with an elliptical beam shape 160 µm (vertical) × 130 µm (horizontal) (FWHM) were provided at 2 Hz repetition rate.

The data collection was interrupted by regular refills of the storage ring every 30 minutes. The data collection software stopped when the X-ray intensity was low, therefore this did not increase the dose on the crystal. The data collection also stopped once during rotation before the exposure started. This did not cause additional radiation damage to the crystal. The diffraction was recorded with a Pilatus 1M detector with a ToT photon-counting mode. The multilayer monochromator selected the photon energy 11.15 keV (Δ*E*/*E* = 0.01) with a flux of 1 × 10^7^ photon pulse^−1^. The beam center was offset from the detector center in order to use the detector surface to collect higher angle diffraction. The program *Raddose3D* was used to estimate the absorbed dose in the crystal (Bury *et al.*, 2018 ▸).

#### 2.1.2. X-ray diffraction data processing of the FemtoMAX data   

The final data set was recovered by merging two wedges of angular range consisting of a total of 1283 images covering 128.3° rotation of the crystal. At the 1283 static position, 100 snapshots were recorded. A Python script was used to sum the 100 snapshots at every rotational position into one image (100× data set). The script used the fabio (Knudsen *et al.*, 2013 ▸), numpy (Walt *et al.*, 2011 ▸) and pandas (McKinney, 2012 ▸) Python libraries. Another data set (1×) was also generated where each image contained only the first snapshot recorded at each rotational position. Thus, the 1× data set does not involve summing of individual snapshots. The images were saved in the CBF (Crystallographic Binary File) (Bernstein & Hammersley, 2005 ▸). The script customized the header of the CBF file to describe the experimental parameters. The images were further processed with the *X-ray Detector Software* (*XDS*) (Kabsch, 2010 ▸). *XDS* was used to process (indexing, refinement, integration, scaling and merging) the images. The programs *pointless* and *aimless* (Evans, 2011 ▸) were employed to determine the *R*_meas_ value as a function of intensity. The Wilson and cumulative intensity distribution plots were generated from the log files of the program truncate of the CCP4 package. The programs aimless and truncate were used only for generating statistical report of the data in Fig. 2. Crystallographic refinement was performed using diffraction intensities generated by the program *XDSCONV*.

#### 2.1.3. Recording the X-ray diffraction image at the BioMAX beamline   

The diffraction image was qualitatively compared with X-ray diffraction data collected from a bovine trypsin crystal at room temperature at BioMAX, a macromolecular crystallography beamline at MAX IV. The crystals were mounted in the MiTiGen plastic capillaries and placed on the mini-kappa goniometer of the beamline. The crystal was exposed to X-rays in shutterless mode while the frames were collected every 11 ms by the Eiger 16M detector.

#### 2.1.4. Structure determination   

An identical set of reflections was used for cross-validation of the data sets (100× and 1×) from FemtoMAX beamline where the resolution range overlapped. The *R*_free_ set was 5% of the total reflections of each data set. The structure was solved with *Phaser* (McCoy, 2007 ▸) of the *PHENIX* software suite (Liebschner *et al.*, 2019 ▸), using the integrated intensities. The refinement was carried out with phenix.refine and the model was manually rebuilt using the software *Coot* (Emsley & Cowtan, 2004 ▸). The refinement was performed with the default options: individual_sites, individual_sites_real_space and individual_adp. All atoms had isotropic atomic displacement parameters and alternative conformations were not modeled. The translation–libration–screw model (Schomaker & Trueblood, 1968 ▸) was not used for any groups of atoms during refinement to describe rigid-body displacements. Riding and free hydrogen atoms were not incorporated in the model.

## 3. Results and discussion   

**Figure 1:**
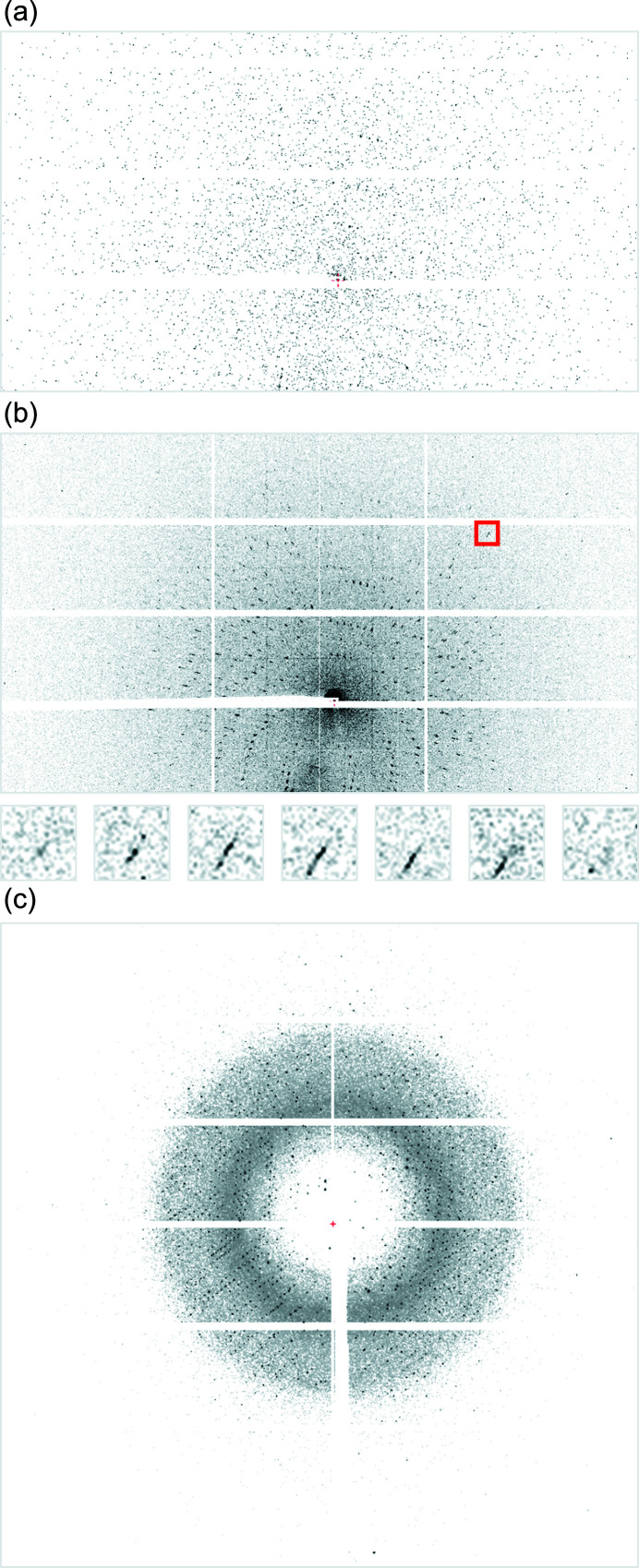
(*a*) A 150 fs snapshot diffraction image from room-temperature bovine trypsin crystal recorded at the FemtoMAX beamline. (*b*) Composite X-ray diffraction image (summing of 100 snapshots) at the FemtoMax beamline. The small images below display a single reflection (red square) on successive images. The position of the Bragg peak changes on the detector at different rotation positions. The recording time at 2 Hz repetition rate is 50 s. (*c*) Summed diffraction of 15 images from room-temperature bovine trypsin crystal recorded at the BioMAX beamline using 1.5 s exposure of 1.5° oscillation angle. The image is zoomed to part of the detector image to reveal the round shape of diffraction spots.

Every diffraction snapshot was associated with a single X-ray pulse and was recorded individually at the FemtoMAX beamline. These images already contained distinguishable Bragg peaks [Fig. 1 ▸(*a*)]. In order to improve the signal-to-noise ratio, the images recorded at the same crystal orientation (100 images per orientation). These images were summed together in the 100× data set, reducing the number of images to one per orientation. The result of the image merging is shown in Fig. 1 ▸(*b*). The diffraction image obtained at the BioMAX beamline from room-temperature bovine trypsin crystal is shown in Fig. 1 ▸(*c*). A comparison of the FemtoMAX and BioMAX images reveals a slightly elongated shape of Bragg peaks [Fig. 1 ▸(*b*)] in the FemtoMAX data as they more radially spread out compared with the BioMAX diffraction spots. Although the elongation is subtle and not noticeable in Fig. 1 ▸(*a*), it is a consequence of the polychromacity of the beam: the inner pixels are activated by higher-energy photons than the outer ones. Nevertheless, the small bandwidth of the X-ray beam limits the extent of streaking, and during data processing we assumed a monochromatic beam. Spatial overlaps were not detected by the data processing programs. The streaking changed the position of the central peak while the reflection was recorded at different positions along the rocking curve [Fig. 1 ▸(*b*)]. This created an additional challenge for profile fitting algorithm in the data processing software *XDS*, but the default parameters were sufficient for successful 3D peak integration.

The dynamic range of the photon-counting detector such as Pilatus is limited when the photons arrive nearly simultaneously to the detector. Count-rate correction is the reason for the potentially reduced dynamic range. This disadvantage is mitigated by the ToT technique where the current is converted to voltage and the duration is measured for which the voltage stays over a predefined threshold. The X-ray photons absorbed in the detector generate a cloud of electrons. This cloud may be bound to a single pixel or is overflown to adjacent pixels. The detected counts are transformed to absorbed energy values based on calibration measurements. Previous tests indicated that the detector can give photon numbers up to 2.5 MeV in a single focused pulse with an error of <10% (Enquist *et al.*, 2018 ▸). A more widely applicable alternative is using integrating detectors such as the recently developed JUNGFRAU detector, which can handle higher photon counts and still maintain high readout rate (Leonarski *et al.*, 2018 ▸).

**Figure 2:**
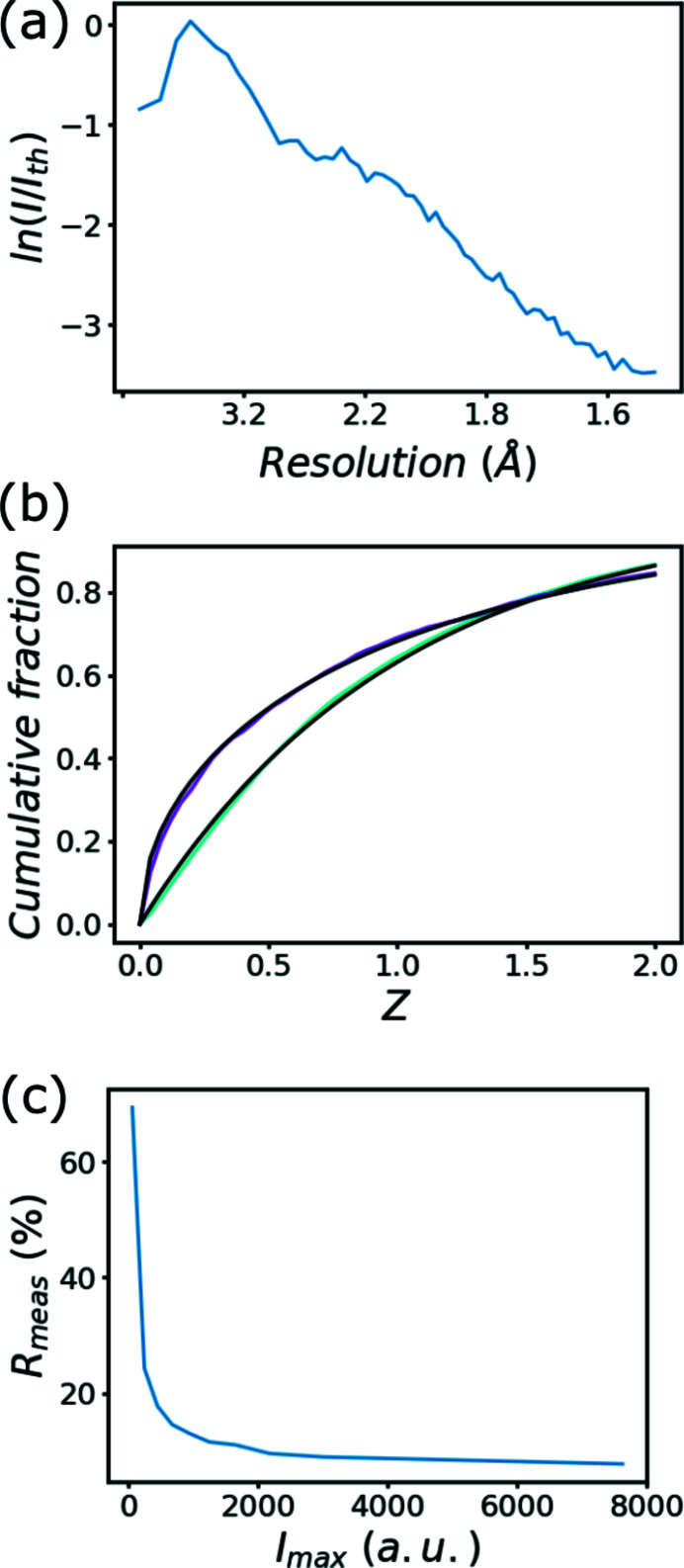
(*a*) Wilson plot of merged reflection intensities for the 100× FemtoMAX data as reported by the program *TRUNCATE* of the CCP4 package. On the *X*-axis 1/*d* ^2^ values are indicated at 0.1, 0.2, 0.3 and 0.4 Å^−2^ and labeled with the equivalent *d* values to aid the intuitive interpretation of the Wilson plot. (*b*) Cumulative intensity distribution plot of acentric (cyan) and centric (magenta) reflections of 100× FemtoMAX data, respectively. The theoretical cumulative intensity distribution is shown in black for both types of reflections. (*c*) *R*_meas_ of symmetry related reflections as a function of intensity.

High-resolution diffraction of protein crystals provides a wide range of diffraction intensities, which can be evaluated by the Wilson plot as function of diffraction angle. Fig. 2 ▸(*a*) shows the Wilson plot recorded at the FemtoMAX beamline. Cumulative intensity distribution offers another way of evaluating reflection intensities. Fig. 2 ▸(*b*) shows the cumulative intensity distribution of acentric and centric reflections, respectively. Both the centric and acentric reflections show very small deviation from (non-twinned) ideality. Fig. 2 ▸(*c*) shows the *R*_meas_ value as a function of reflection intensity. The highest-intensity reflections show the smallest *R*_meas_ values, because measurement errors in these reflections tend to contribute less to intensity observations. If these strong reflections would have an increased *R*_meas_ value this could indicate dynamic range issues. Additional data statistics are listed in the supporting information as reported by the program *XSCALE*. Bovine trypsin crystals at room temperature provide some of the highest intensity and most focused diffraction (due to low mosaicity). This could be a problem because the detector pixels even in the ToT mode tolerate only a limited number of simultaneous photon absorptions. If the crystal quality is high, the crystals themselves do not enlarge the Bragg peaks very much and the reflections could overload the detector pixels. Most protein crystals have lower diffracting power and higher mosaicity than bovine trypsin crystals resulting in less intense and larger Bragg peaks. Therefore, the choice of protein system is not likely limited by the ToT mode of the detector with the current experimental parameters of the FemtoMAX beamline. It is important to note that many protein crystals have much lower diffracting power and the low average photon flux of the beamline will set the limit to the suitability of the system instead. The total maximum absorbed dose and average dose was approximately 27 kGy and 7 kGy, respectively, during the 18 h of data collection. This absorbed dose is well below the room-temperature Garman limit of many protein crystals (400–100 kGy) (Atakisi *et al.*, 2019 ▸; Leal *et al.*, 2013 ▸). The long data collection may lead to dehydration of more sensitive protein crystals. This risk can be partially mitigated by the upgrade of the FemtoMAX beamline to 100 Hz repetition rate, which allows 50-fold reduction of the data collection time without sacrificing the data quality. Cryogenic cooling of suitable samples can prevent dehydration entirely and simultaneously improve radiation damage tolerance of the crystals.

**Figure 3:**
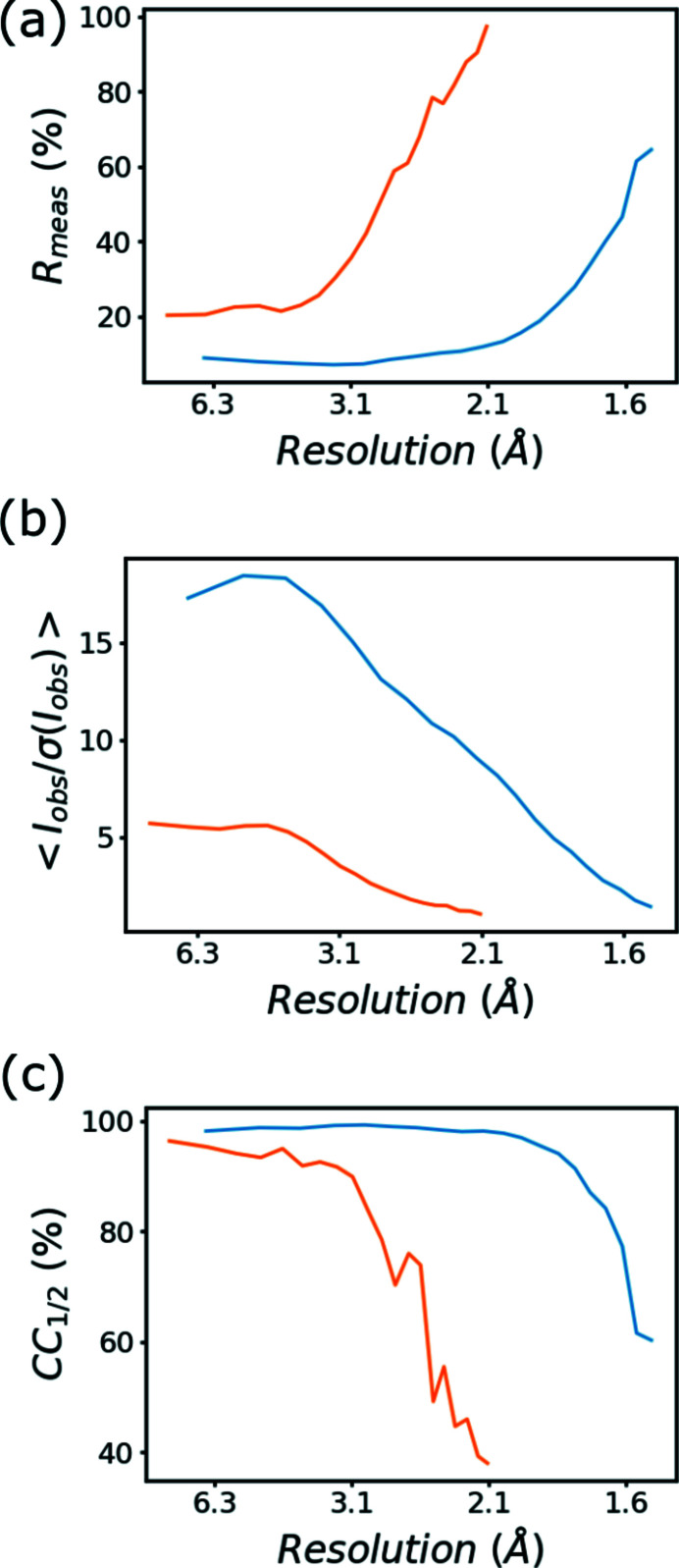
(*a*) *R*_meas_, (*b*) mean *I*/σ(*I*) and (*c*) CC_1/2_ values as a function of resolution for the 100× FemtoMAX (blue) and 1× FemtoMAX (orange) data, respectively. The *X*-axis indicates the upper limit of the resolution bins as reported by the program *XSCALE*.

The standard statistical description of the 100× and 1× data sets is listed in Table 1 ▸ and in the supporting information. The gain value reflects that the ToT measurements return photon energy in keVs, and the gain corresponds to the energy of one photon in keV. By using this gain the counting uncertainty is better estimated. The mosaicity and beam divergence are relatively high. This is a direct consequence of the polychromacity of the beam, and the integration software *XDS* does not have specific model parameters for modeling the X-ray spectrum. Instead, the apparent broadening of reflections is compensated by these two parameters. It is also easier to notice the clustering of high-valued pixels when the diffraction image from single X-ray pulses are observed. For the 1× data set the default parameters for spot picking and indexing were sufficient for identifying the unit-cell parameters and crystal orientation. The quality indicators *R*_meas_, 〈*I*/σ(*I*)〉 and CC_1/2_ as a function of resolution bins are displayed in Fig. 3 ▸ for both data sets. The overall *R*_meas_ statistics are substantially better in the 100× data set even for the lowest resolution bins as a result of low number of photon counts. There is a similar trend in the mean *I*/σ(*I*) statistics, which, except for the lowest-resolution bin, monotonously decreases in both data sets. The CC_1/2_ statistics start to decrease in the 1× data sets immediately; in the 100× it becomes noticeable at around 2.1 Å resolution.

**Figure 4:**
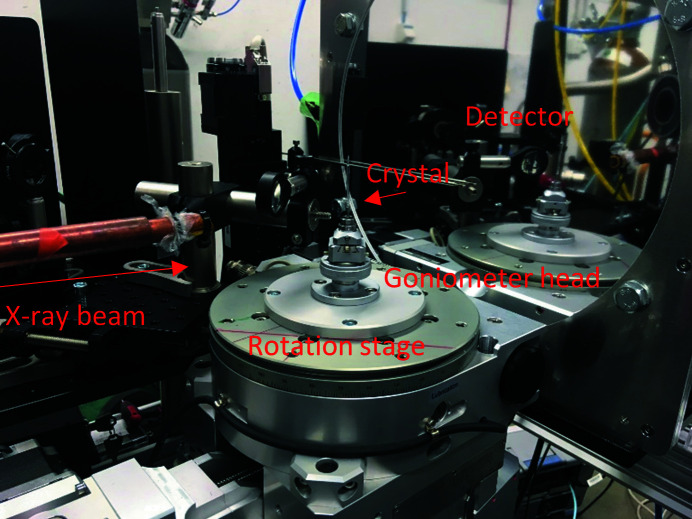
The macromolecular crystallography experimental setup at the FemtoMAX beamline.

The overall completeness is lower than what one could expect for an orthorhombic crystal system after 128.3° rotation (Table 1 ▸). Firstly, the beam was offset from the detector center. Secondly, the aspect ratio of the detector was not 1.0. Thirdly, some reflections were only observed in the two upper corners of the detector. The steep drop in completeness is due to the third point. The achievable shortest detector distance was limited by the spatial extent of the crystal rotation stage (Fig. 4 ▸) and the highest-resolution reflections were only observed in the upper corners of the detector.

**Figure 5:**
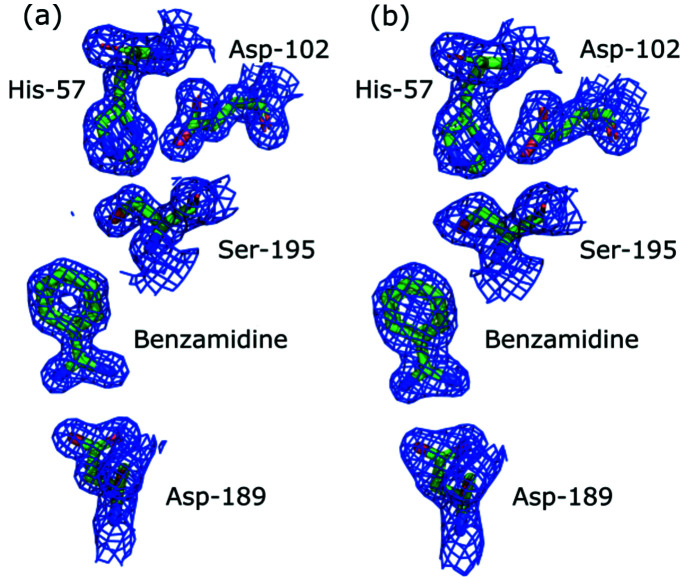
2mF_o_ − DF_c_ electron density was recovered from (*a*) 100× and (*b*) 1× FemtoMax data. The missing structure factor amplitudes were not replaced with calculated structure factors. The figure shows the catalytic triad and the bound aromatic benzamidine inhibitor. The 2mF_o_ − DF_c_ electron density maps were contoured at the 1.5σ level.

We performed crystallographic refinement in order to describe the quality of the data. Molecular replacement and refinement were straightforward for both data sets. After iterative refinement-rebuilding cycles the final *R*_free_ of the 100× and 1× models were 19.4% and 26.0%, respectively. The recovered 2mF_o_ − DF_c_ electron density showed fine details in the 100× data set, and the contours of amino acid residues are still accurate in the 1× data set (Fig. 5 ▸). In the 100× electron density maps, the lower electron density in the middle of aromatic rings such as in the bound benzamidine inhibitor is clearly visible. Average isotropic *B*-factors of the atoms relate to the Wilson *B*-factor in both cases similarly: Wilson *B*-factors are approximately 5 Å^2^ lower than the average modeled isotropic *B*-factors. The overall maximum likelihood-based coordinate error estimate is 0.18 Å and 0.31 Å for the 100× and 1× crystallographic models, respectively.

## 4. Conclusion   

In this short report, we have demonstrated the feasibility of performing protein crystallographic experiments at the FemtoMAX beamline of the MAX IV synchrotron. We showed that the potential problems of limited flux of the beamline, the simultaneous arrival of diffracted photons on the photon-counting area detector and the use of a multilayer monochromator have limited influence on the data quality in practice. The maximum attainable resolution of bovine trypsin crystals was 1.5 Å when 100 ultrafast snapshots were summed. This level of detail rivals the best data obtained at XFELs already. The diffraction data can be processed without additional efforts from single snapshots, and the model quality is still acceptable at the attained 2.1 Å resolution. We anticipate that the signal-to-noise ratio will increase further for an equivalent data collection time when the beamline will be upgraded to 100 Hz repetition rate.

## Supplementary Material

Crystal data. DOI: 10.1107/S1600577520014599/wz5014sup1.mcf

Extract of the XSCALE log file. DOI: 10.1107/S1600577520014599/wz5014sup2.pdf

## Figures and Tables

**Table 1:** Room-temperature data collection and refinement statistics of bovine trypsin crystals at beamline FemtoMAX

	100× summation	No summation (1×)
Experimental parameters		
Wavelength (Å)	1.112	
Bandwidth	0.01	
Detector distance (mm)	142.0	
Rotation step/oscillation range (°)	0.1	
Mean flux (photons s^−1^)	2 × 10^7^	
Exposure time per frame	150 fs	
Photons per dataset	1.3 × 10^12^	1.3 × 10^10^
Approximate collection time	18 h	3.5 h

Diffraction data statistics		
Resolution range (Å)	42.56–1.50 (1.54–1.50)	42.56–2.10 (2.15–2.10)
Space group	*P*2_1_2_1_2_1_	*P*2_1_2_1_2_1_
Unit-cell dimensions	54.84 Å, 58.70 Å, 67.47 Å, 90°, 90°, 90°	54.84 Å, 58.70 Å, 67.47 Å, 90°, 90°, 90°
Total reflections	72494 (1573)	45677 (2755)
Unique reflections	28730 (1079)	12533 (879)
Gain	11.15	
Reflecting range (mosaicity) (°)	0.152	0.120
Beam divergence (°)	0.096	0.071
Multiplicity	2.5 (1.5)	3.6 (3.1)
Completeness (%)	80.8 (41.6)	95.0 (91.8)
Mean *I*/σ(*I*)	8.0 (1.4)	2.8 (1.1)
Wilson *B*-factor (Å^2^)	14.6	21.0
*R*-merge (%)	7.7 (47.5)	31.0 (83.0)
*R*-meas (%)	9.1 (64.5)	35.5 (97.4)
CC_1/2_ (%)	99.3 (60.3)	93.1 (38.0)

Refinement statistics		
Resolution range (Å)	42.56–1.50 (1.55–1.50)	42.56–2.10 (2.18–2.10)
Reflections used in refinement	28724 (1544)	12524 (1153)
Reflections used for *R*-free	1445 (89)	628 (59)
*R*-work	0.1641 (0.3077)	0.2128 (0.3138)
*R*-free	0.1937 (0.3584)	0.2602 (0.3593)
Number of non-hydrogen atoms	1822	1800
Macromolecules	1629	1629
Ligands	15	15
Solvent	178	156
Protein residues	223	223
RMS (bonds) (Å)	0.005	0.009
RMS (angles) (°)	0.77	1.00
Ramachandran favored (%)	98.6	97.3
Ramachandran allowed (%)	1.4	2.7
Ramachandran outliers (%)	0	0
Rotamer outliers (%)	0	0
Clashscore	1.23	2.16
Average *B*-factor (Å^2^)	19.7	26.0
Macromolecules	18.6	25.5
Ligands	23.0	28.0
Solvent	29.7	31.1
PDB entry	6XYG	7AYS

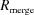
 = 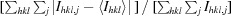. 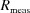 = 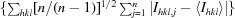/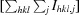, where *hkl* refers to the Miller index of the reflection, *j* the specific observation. 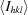 is the average of all intensity observations.

Molecular coordinates and structure factor amplitudes are available through the Protein Data Bank (accession codes 6xyg and 7ays) and the diffraction images have been deposited in the Zenodo data base (doi:10.5281/zenodo.4290178).
