# Supplementary material for: High-resolution macromolecular crystallography at the FemtoMAX beamline with time-over-threshold photon detection
Source: J Synchrotron Radiat. 2021 Jan 1;28(Pt 1):64–70. doi: 10.1107/S1600577520014599 (PMC7842217; doi:10.1107/S1600577520014599)
Supplement: Supplementary file 2 [file s-28-00064-sup2.pdf]

JOURNAL OF SYNCHROTRON RADIATION

ISSN 1600-5775

Accepted article (4 November 2020)

High-resolution macromolecular crystallography at the FemtoMAX beamline with time-over-threshold photon detection

Maja Jensen,[a ‡] Viktor Ahlberg Gagnér,[a ‡] Juan Cabello Sánchez,[b] Åsa U. J. Bengtsson,[c] J. Carl Ekström,[c] Tinna Björg Úlfarsdóttir,[a] Maria-Jose Garcia-Bonete,[a] Andrius Jurgilaitis,[d] David Kroon,[d] Van-Thai Pham,[d,e] Stefano Checcia,[d] Hélène Coudert-Alteirac,[d] Siawosch Schewa,[f] Manfred Rössle,[f] Helena Rodilla,[b] Jan Stake,[b] Vitali Zhaunerchyk,[g] Jörgen Larsson[c] and Gergely Katona[a *]

[a]Department of Chemistry and Molecular Biology, University of Gothenburg, Gothenburg, Sweden, [b]Department of Microtechnology and Nanoscience, Chalmers University of Technology, Gothenburg, Sweden, [c]Department of Physics, Lund University, PO Box 118, Lund, 22100, Sweden, [d]MAX IV Laboratory, Lund University, PO Box 118, Lund, 22100, Sweden, [e]Center for Quantum Electronics, Institute of Physics, Vietnam Academy of Science and Technology, Hanoi, Vietnam, [f]Technische Hochschule Lübeck, Lübeck, Germany, and [g]Department of Physics, University of Gothenburg, Gothenburg, Sweden

[*] Correspondence email: gergely.katona@gu.se

[‡] These authors contributed equally to this work.

Keywords: time-over-threshold; femtosecond; multilayer monochromator; macromolecular crystallography

Funding information: Rontgen-Angstrom Cluster Framework (award No. 2015-06099)

Table S1 Extract from the XSCALE log file displaying the resolution dependent intensity statistics.

100x data set

SUBSET OF INTENSITY DATA WITH SIGNAL/NOISE >= -3.0 AS FUNCTION OF RESOLUTION

RESOLUTION LIMIT	NUMBER OF REFLECTIONS OBSERVED	UNIQUE	POSSIBLE	COMPLETENESS OF DATA	R-FACTOR observed	R-FACTOR expected	COMPARED	I/SIGMA	R-meas	CC(1/2)	Anomal Corr	SigAno	Nano
6.71	1333	421	461	91.3%	7.7%	7.4%	1250	17.27	8.9%	98.2*	-9	0.818	151
4.74	2832	765	784	97.6%	6.9%	7.5%	2746	18.42	7.9%	98.8*	-4	0.777	357
3.87	3260	944	984	95.9%	6.4%	7.3%	3139	18.29	7.4%	98.7*	-15	0.672	402
3.35	3629	1090	1152	94.6%	6.2%	7.5%	3489	16.88	7.1%	99.2*	-18	0.643	424
3.00	3772	1201	1289	93.2%	6.2%	7.6%	3629	15.03	7.3%	99.3*	-13	0.700	388
2.74	4182	1334	1430	93.3%	7.2%	8.1%	4022	13.11	8.5%	99.0*	-14	0.711	466
2.54	4240	1397	1524	91.7%	7.8%	8.4%	4059	12.08	9.3%	98.8*	-14	0.726	450
2.37	4400	1496	1644	91.0%	8.5%	9.1%	4175	10.84	10.2%	98.4*	-11	0.761	429
2.24	4294	1521	1736	87.6%	8.9%	9.4%	4030	10.16	10.7%	98.1*	-10	0.765	381
2.12	4422	1623	1828	88.8%	9.9%	10.2%	4125	9.07	11.9%	98.2*	-2	0.833	361
2.02	4418	1702	1926	88.4%	10.9%	11.6%	4050	8.17	13.3%	97.8*	-12	0.786	323
1.94	4385	1765	2019	87.4%	12.8%	13.3%	3960	7.16	15.6%	97.0*	-15	0.794	283
1.86	4335	1825	2105	86.7%	15.2%	15.2%	3867	5.90	18.8%	95.5*	-11	0.773	240
1.79	4228	1855	2173	85.4%	18.6%	18.9%	3704	4.92	23.3%	94.1*	-18	0.705	203
1.73	4136	1897	2223	85.3%	22.1%	22.8%	3574	4.25	27.9%	91.4*	5	0.825	163
1.68	3953	1888	2321	81.3%	26.2%	64.0%	3344	3.49	33.6%	87.1*	6	0.861	134
1.63	3646	1827	2397	76.2%	31.0%	36.8%	2991	2.78	40.0%	84.2*	-8	0.731	101
1.58	3119	1682	2462	68.3%	35.6%	38.2%	2421	2.31	46.5%	77.3*	1	0.792	46
1.54	2337	1418	2517	56.3%	45.8%	58.7%	1617	1.76	61.4%	61.6*	-8	0.641	13
1.50	1573	1079	2591	41.6%	47.5%	80.6%	906	1.44	64.5%	60.3*	-19	0.789	10
total	72494	28730	35566	80.8%	7.7%	8.8%	65098	8.00	9.1%	99.3*	-10	0.747	5325

1x data set

SUBSET OF INTENSITY DATA WITH SIGNAL/NOISE >= -3.0 AS FUNCTION OF RESOLUTION

RESOLUTION LIMIT	NUMBER OF REFLECTIONS OBSERVED	UNIQUE	POSSIBLE	COMPLETENESS OF DATA	R-FACTOR observed	R-FACTOR expected	COMPARED	I/SIGMA	R-meas	CC(1/2)	Anomal Corr	SigAno	Nano
9.39	597	169	181	93.4%	17.7%	24.5%	571	5.71	20.3%	96.4*	-21	0.551	61
6.64	1184	297	298	99.7%	18.2%	26.8%	1150	5.52	20.5%	95.3*	3	0.724	137
5.42	1568	359	363	98.9%	19.9%	37.5%	1545	5.42	22.5%	94.1*	1	0.752	196
4.70	1939	434	435	99.8%	20.2%	32.7%	1920	5.58	22.8%	93.4*	-15	0.639	242
4.20	1950	470	479	98.1%	18.9%	29.8%	1905	5.60	21.4%	95.0*	6	0.754	240
3.83	2101	514	536	95.9%	20.1%	36.0%	2053	5.27	23.0%	91.9*	8	0.786	264
3.55	2222	550	569	96.7%	22.5%	44.7%	2167	4.76	25.6%	92.6*	11	0.777	280
3.32	2276	599	619	96.8%	26.7%	56.6%	2211	4.14	30.4%	91.7*	-4	0.773	281
3.13	2335	625	647	96.6%	31.2%	73.2%	2264	3.51	35.7%	89.9*	-4	0.692	273
2.97	2395	635	664	95.6%	37.0%	92.7%	2329	3.10	42.2%	84.0*	-7	0.667	297
2.83	2567	694	718	96.7%	44.2%	122.8%	2492	2.63	50.7%	78.5*	2	0.780	314
2.71	2656	716	744	96.2%	51.5%	126.7%	2575	2.32	58.8%	70.3*	-7	0.704	331
2.60	2673	740	773	95.7%	53.0%	161.4%	2590	2.04	60.9%	76.0*	-14	0.632	330
2.51	2735	758	801	94.6%	59.1%	183.7%	2648	1.80	68.0%	73.9*	2	0.692	343
2.42	2726	781	825	94.7%	67.7%	205.0%	2632	1.62	78.4%	49.2*	-3	0.669	316
2.35	2731	800	853	93.8%	66.5%	208.9%	2618	1.51	76.8%	55.5*	-6	0.630	315
2.28	2738	817	887	92.1%	70.3%	209.8%	2621	1.49	81.8%	44.7*	-2	0.709	314
2.21	2805	843	915	92.1%	75.2%	213.1%	2706	1.23	87.9%	46.0*	0	0.678	285
2.15	2724	853	930	91.7%	77.2%	248.8%	2600	1.21	90.4%	39.3*	3	0.622	275
2.10	2755	879	957	91.8%	83.0%	301.9%	2635	1.06	97.4%	38.0*	8	0.684	260
total	45677	12533	13194	95.0%	31.0%	73.9%	44232	2.76	35.5%	93.1*	-1	0.699	5354
